# Perioperative care following complex laryngotracheal reconstruction in infants and children

**DOI:** 10.4103/1658-354X.71577

**Published:** 2010

**Authors:** Punkaj Gupta, Joseph D. Tobias, Sunali Goyal, Sana F. Hashmi, Jennifer Shin, Christopher J. Hartnick, Natan Noviski

**Affiliations:** 1 *Division of Pediatric Critical Care, Massachusetts General Hospital, Harvard Medical School, Boston, MA, USA*; 2 *Lucile Packard Children’s Hospital, Stanford University Medical Center, Palo Alto, CA, USA*; 3 *Departments of Anesthesiology and Pediatrics, Nationwide Children’s Hospital and the Ohio State University, Columbus, OH, USA*; 4 *Department of Medical Education, Metrowest Medical Center, Framingham, MA, USA*; 5 *Stanford University School of Medicine, Palo Alto, CA, USA*; 6 *Department of Otolaryngology, Massachusetts Eye and Ear Infirmary, Harvard Medical School, Boston, MA, USA*

**Keywords:** *Extubation*, *laryngotracheal reconstruction*, *reintubation*, *tracheostomy*

## Abstract

Laryngotracheal reconstruction (LTR) involves surgical correction of a stenotic airway with cartilage interpositional grafting, followed by either placement of a tracheostomy and an intraluminal stent (two-stage LTR) or placement of an endotracheal tube with postoperative sedation and mechanical ventilation for an extended period of time (singlestage LTR). With single-stage repair, there may be several perioperative challenges including the provision of adequate sedation, avoidance of the development of tolerance to sedative and analgesia agents, the need to use neuromuscular blocking agents, the maintenance of adequate pulmonary toilet to avoid perioperative nosocomial infections, and optimization of postoperative respiratory function to facilitate successful tracheal extubation. We review the perioperative management of these patients, discuss the challenges during the postoperative period, and propose recommendations for the prevention of reversible causes of extubation failure in this article. Optimization to ensure a timely tracheal extubation and successful weaning of mechanical ventilator, remains the primary key to success in these surgeries as extubation failure or the need for prolonged postoperative mechanical ventilation can lead to failure of the graft site, the need for prolonged Pediatric Intensive Care Unit care, and in some cases, the need for a tracheostomy to maintain an adequate airway.

## INTRODUCTION

Laryngotracheal reconstruction (LTR) involves surgical correction of a stenotic airway, generally in the subglottic region, with cartilage interpositional grafting. This is followed by either placement of a tracheostomy tube and an intraluminal stent (two-stage LTR) or the use of an endotracheal tube (ETT) to act as a stent (single-stage LTR).[1–3] This procedure was first introduced by Fearon and Cotton in 1972 with the aim of expanding stenotic airway segments in children with congenital and acquired laryngotracheal stenosis.[4] Single-stage LTR presents particular challenges to the perioperative team of pediatric otolaryngologists, intensive care specialists, and pediatric anesthesiologists due to the requirements of providing adequate analgesia and sedation for a prolonged period of time to allow the graft to heal and subglottic edema to resolve while attempting to minimize the sequelae of the prolonged use of sedative and analgesic agents. Additionally, in some circumstances, the risk of iatrogenic morbidity may be further increased by the need for neuromuscular blocking agents (NMBAs) when adequate sedation cannot be maintained. The postoperative management of these patients can be complicated by airway and respiratory issues including pulmonary atelectasis, nosocomial pneumonia, airleak syndromes, leakage at the tracheal graft site, and post-extubation airway obstruction.[1] Of primary importance is avoidance of inadvertent tracheal extubation as well as the identification of the appropriate time for tracheal extubation, as airway manipulation or prolonged endotracheal intubation may result in surgical failure, prolonged Pediatric Intensive Care Unit (PICU) care, and in some cases, the need for a tracheostomy to maintain an adequate airway.[2] With single-stage repair, there may be several postoperative challenges including the provision of adequate sedation, avoidance of the development of tolerance to sedative and analgesia agents, the need to use NMBAs, the maintenance of adequate pulmonary toilet to avoid perioperative nosocomial infections, optimization of postoperative respiratory function to facilitate successful and early tracheal extubation, and the prevention of dysphagia and aspiration when the stent is in place.

The postoperative management of these patients requires a comprehensive understanding of the principles of airway reconstruction and extensive experience on the part of the surgeon, anesthesiologist, pediatric intensive care physician, respiratory therapist and the nursing staff for a successful outcome. We discuss in this article the challenges during the postoperative period and identify certain techniques that may be used during the perioperative period, which may decrease the morbidity associated with this complex surgery in this tenuous age group.

Following single-stage LTR, young and uncooperative children generally require significant sedation and analgesia to control agitation and prevent inadvertent tracheal extubation. In some centers, NMBAs are also administered to ensure immobility and decrease the risk of graft failure. Additional pharmacologic therapy which may impact on the perioperative course includes the administration of corticosteroids for 2–5 days to limit airway and surgical site edema. Given both the morbidity imposed by the surgical procedure, requirements during the postoperative course for sedation and pharmacologic paralysis, as well as associated co-morbidities, the perioperative care of such patients can be challenging. Given the risks associated with airway instrumentation including laryngoscopy and tracheal intubation, the primary goal of the perioperative care of such patients is to optimize their physiologic status in the hopes of decreasing the incidence of extubation failure. The etiology of extubation failure may be categorized as reversible (presence of granulation tissue, mucosal edema, excessive secretions, sedative withdrawal, neuromuscular weakness) or irreversible (prolapsed grafts and/or restenosis of the airway site).[5,6] When determining protocols for the perioperative care of such patients, the following factors should be considered.

## ROUTE OF ENDOTRACHEAL INTUBATION

Although the oral route is generally chosen for endotracheal intubation in the PICU population, in this select patient population, various studies have suggested that there may be advantages of using the nasal route especially when prolonged endotracheal intubation (3–7 days) is anticipated in the PICU setting.[1–3] Although there are no prospective, randomized trials comparing the oral versus the nasal route for endotracheal intubation in this patient population, various authors have emphasized the potential advantages of the nasal route.[1–3] In a series of 38 pediatric patients following LTR, Yellen *et al*. recommended that the nasal route be used.[3] All 21 patients who underwent single-stage LTR were nasotracheally intubated for a mean of 8.4 days (range 6–14 days) with an overall success rate of 95%.[3] Although there are no prospective, randomized trials in either the adult or pediatric population to demonstrate the clinical advantages of the nasal versus the oral route for endotracheal intubation in the ICU setting, our clinical impression and that of others is that the nasal route provides more secure control of the airway with a decreased risk of inadvertent extubation and decreases sedation requirements, leading to faster recovery and shorter PICU as well as hospital stay.[1–3] The nasal route also allows for easier and more efficient mouth care. The latter may be especially important during protracted ICU stays as recent data suggest that topical use of oral chlorhexidine may help decrease the incidence of nosocomial infections.[7] The use of nasotracheal intubation during surgery may also maximize surgical exposure of the surgical field during complex repairs. Hall *et al*. advocated the use of nasotracheal intubation in this select patient population as the benefits of nasotracheal intubation to the head and neck surgeon outweigh the potential disadvantages.[8] Oro-tracheal tubes can be obstructed by biting and more readily displaced by tongue movement and gagging in awake patients.[8,9]

Despite such advantages, there are certain caveats when choosing the nasotracheal route as the preferred route for endotracheal intubation. The ETT should be one size smaller than that generally used based on the patient’s age, as larger tubes may damage the nares, displace adenoidal tissue or lead to epistaxis during tube placement. Consideration should be given to the use of smaller cuffed ETTs, as minimal inflation of the cuff may be needed to ensure adequate sealing of the airway to allow for effective tidal volumes. Smaller uncuffed ETTs may lead to excessive leaks and interfere with effective positive pressure mechanical ventilation. Recent advances in the design of cuffed pediatric ETTs have resulted in the manufacturing of cuffed ETTs with a low profile, low pressure cuff. Studies from both the operating room and the ICU demonstrate no difference in postoperative complications when comparing cuffed versus uncuffed ETTs with a decrease in the need to change cuffed tubes due to size issues and the inability to effectively seal the airway.[10,11] Additional caveats to consider when using cuffed ETTs is to slowly inflate the cuff with the minimal amount of air necessary to seal the airway and to frequently check the cuff pressure to ensure that pressures are kept ≤20 cm H_2_O to avoid compromising tracheal perfusion pressure, thereby limiting postoperative subglottic issues.

As nasotracheal intubation may be time-consuming to allow for airway preparation, many practitioners choose to provide orotracheal intubation and then switch to the nasotracheal route. Other means to limit the potential trauma to the nares and nasopharynx during placement of the ETT include the administration of a topical vasoconstricting agent (Afrin^®^ nasal spray), passage of progressively larger nasopharyngeal airways which are lubricated with a topical agent such as 2% lidocaine jelly (a vasoconstrictor such as phenylephrine can be added to the lidocaine jelly), and warming of the ETT prior to use. The latter may be accomplished by placing the ETT in warm water for 20–30 minutes prior to use. Additionally, Elwood *et al*. suggested placing a red rubber catheter over the end of the ETT prior to its passage through the nasopharynx to avoid damage and bleeding during passage of the ETT.[12]

## SEDATION

Following LTR, continuous sedation has been advocated in infants and young children to avoid movement and trauma from the ETT against the fresh graft site and avoid the potentially life-threatening complication of inadvertent tracheal extubation.[5] Sedative and analgesic agents, although mandatory following surgical procedures and during ongoing endotracheal intubation, may result in adverse respiratory effects including depressed cough reflex, ineffective clearing of secretions, diminished sigh volumes, decreased functional residual capacity as well as hemodynamic effects which may not be well tolerated in the immediate postoperative period or in infants with co-morbid disease processes. Following the prolonged administration of these agents, their abrupt discontinuation may result in a withdrawal syndrome which may complicate the process of tracheal extubation. Despite the beneficial effects of adequate sedation, tolerance and physical dependency to these medications develops in up to 50–60% of patients.[13] Treatment strategies and protocols are necessary so that the problems of tolerance, physical dependency, and withdrawal do not limit the administration of these agents in this select patient population. The manifestations of withdrawal vary according to the agent used for sedation, manifesting shortly after discontinuation of the drug if the agent has a short half-life (propofol, fentanyl or morphine) or days later if the agent or its metabolites have long half-lives (diazepam).[13] As tolerance develops related to receptor occupancy, it is theoretically possible to delay its development by using agents with decreased agonism at the receptor or by the use of rotating sedation regimens at specific intervals. One of the other strategies suggested in patients receiving long-term continuous sedation can be replacing synthetic opioids such as fentanyl with non-synthetic opioids such as morphine, as synthetic opioids have increased affinity for the opioid receptor, which may result in tolerance more rapidly and a higher incidence of withdrawal than non-synthetic opioids.[13,14] In a study comparing the effects of morphine and fentanyl on the prevalence of withdrawal after extracorporeal membrane oxygenation, Franck *et al*. demonstrated that neonates receiving morphine required less supplemental analgesia than did neonates who received fentanyl and had a significantly lower incidence of withdrawal.[15] This resulted in a more rapid hospital discharge in neonates receiving morphine than those who had received fentanyl. Other potential advantages of morphine are illustrated by additional reports demonstrating not only efficacy, but also beneficial physiologic effects.[16,17] Lynn *et al*. demonstrated that morphine infusions of 10–30 µg/kg/hour provided effective analgesia without affecting weaning from mechanical ventilation following cardiac surgery in neonates and infants.[16] Morphine infusions are also effective in blunting the sympathetic stress response and reducing epinephrine adrenaline levels during mechanical ventilation.[17] In their study of 41 mechanically ventilated babies who were treated with surfactant for hyaline membrane disease, Quinn *et al*. demonstrated that morphine-treated neonates had a significant reduction in plasma epinephrine concentrations without adverse hemodynamic effects.[17]

To date, there are limited data to demonstrate the efficacy of a rotating sedation regimen in preventing tolerance and thereby limiting the incidence of withdrawal. Wheeler *et al*. presented preliminary data on a strategy employing a rotating sedation regimen, derived from the treatment of two patients who required sedation following LTR.[14] The sedation regimen included a midazolam infusion (0.1–0.15 mg/kg/hour) with as needed doses of morphine on day 1, a fentanyl infusion (2–3 µg/kg/hour) with as needed doses of lorazepam on day 2, and a dexmedetomidine infusion (0.25–0.3 µg/kg/hour) with as needed doses of morphine on day 3. The day 1 regimen was repeated on day 4, the day 2 regimen on day 5, and the day 3 regimen on day 6 so that tracheal extubation occurred during the dexmedetomidine infusion. The authors compared the development of tolerance, physical dependency, withdrawal and hospital discharge in the two patients treated with a rotating sedation regimen with five patients who received the conventional regimen of a continuous infusions of midazolam and intermittent doses of morphine for their entire 5 day postoperative course following LTR. The authors noted no clinical signs or symptoms of withdrawal in the two patients treated with the rotating sedation regimen, whereas all five patients who received continuous infusion of a single agent manifested either mild or moderate withdrawal. The problems of tolerance and withdrawal manifested by the patients who had not received the rotating regimen resulted in the need for a longer hospital stay (mean of 8.6 days versus 6.5 days) and a delay in the time for the resumption of full oral fluid intake.

In addition to issues such as tolerance, withdrawal, and physical dependency which may lead to problems following tracheal extubation, the prolonged effects of sedative and analgesic agents may compromise upper airway control and ventilator function, leading to respiratory failure following tracheal extubation. Patients less than 4 years of age are more vulnerable to prolonged sedation, with a resultant increase in failure rates following tracheal extubation.[2] Although short-acting agents such as fentanyl and midazolam are frequently chosen for sedation during mechanical ventilation, significant changes in their elimination half-life may occur with prolonged administration (context-sensitive half-life) so that a prolonged effect is noted following their discontinuation.

One of the means of limiting residual sedation includes titration of infusion rates to the desired level of sedation by following pediatric pain scores.[18] The currently used PICU sedation scores evaluate either physiologic variables such as heart rate and blood pressure, an objective assessment of the patient’s depth of sedation, or a combination of the two. One commonly used scale, the COMFORT score, combines the scoring of a patient’s response or movement in addition to various physiologic parameters.[19] It relies on the measurement of alertness, respiration, blood pressure, muscle tone, agitation, movement, heart rate, and facial tension. This scoring system has been validated in the pediatric-aged patient and may have utility in the assessment of sedation during mechanical ventilation.[19,20] However, scales that use physiologic parameters can be misleading in an ICU setting where alterations in vital signs can occur unrelated to the level of sedation. Furthermore, patients with cardiovascular dysfunction requiring vasoactive medications may not manifest increases in heart rate and blood pressure even in the presence of severe agitation or pain. Because of these concerns, Ista *et al*. have recently proposed a modified original COMFORT score, known as the COMFORT-B score which eliminates the use of physiologic variables and provides new cutoff points for the diagnosis of oversedation or undersedation.[21] Other scoring systems, such as the Sedation-Agitation Scale (SAS), also eliminate the use of physiologic parameters. The SAS visually assesses the level of the patient’s comfort and grades it from 1 (unarousable) to 7 (dangerous agitation such as pulling at the ETT).[22] The Ramsay Scale, a sedation scale used commonly in the adult ICU population, not only assigns a value based on the observation of the patient but also uses a tactile stimulus (a glabellar tap) to distinguish between the deeper levels of sedation.[23] Scoring for the Ramsey score varies from 1 (awake, anxious and agitated) to 6 (no response to a glabellar tap). The Hartwig score similarly uses a visual assessment of the patient, but as with the Ramsay scale, includes a response to a noxious stimulus (in this case, tracheal suctioning), thereby eliminating its use in non-intubated patients.[24] Scales such as the Ramsay and the Hartwig that assess the response to a tactile stimulus require disturbing the patient to differentiate between the deeper levels of sedation. Additionally, scales that evaluate a patient’s response to a stimulus or observe their behavior are not valid during the administration of NMBAs which prevent movement.

As none of these sedation scales meets all of the needs of the PICU provider, there remains an interest in the use of monitoring technology which may be able to assess the depth of sedation through the analysis of the electroencephalogram (EEG). The Bispectral Index (BIS monitor) (Aspect Medical, Newton, MA, USA) uses a programmed algorithm to evaluate the processed EEG pattern and provide a numeric value ranging from 0 (isoelectric) to 100 (awake with eyes open). Its predominant clinical use has been intraoperatively to monitor the effects of general anesthetic and sedative agents and provide a measure of the depth of anesthesia. Although still somewhat controversial, it has been suggested that maintenance of a BIS value to less than 60–70 correlates with a low probability of intraoperative awareness.[25,26] Caveats regarding the use of BIS are that the algorithm was developed during the use of general anesthetic agents such as propofol, barbiturates, benzodiazepines or the inhalational anesthetic agents which act through the γ-amino butyric acid (GABA) system. Additionally, as there are differences between the EEG of the adult and children less than 6-8 years of age, these devices may have limited utility in younger patients. Although the results have been somewhat mixed, the majority of reports have demonstrated a clinically acceptable correlation between the BIS monitor and commonly used ICU sedation scores including the Ramsay or the COMFORT score.[27–31] Although clinical pain scores are generally quite useful to allow for the effective titration of the sedation regimen, when pain scores are not feasible such as during the use of NMBAs, processed EEG monitoring may be helpful in the evaluation of the depth of sedation.

Additional means of limiting the residual effects of sedative and analgesic agents during prolonged infusions in the PICU include the use of intermittent dosing rather than a continuous infusion or the use of “drug holidays” as have been popular in the care of adult ICU patients. In a landmark study by Kress *et al*., daily interruption of sedative drug infusions decreased the duration of mechanical ventilation and the length of stay in the intensive care medicine in their cohort of 128 patients.[32] The median duration of ventilation and median length of stay in ICU was significantly reduced in the interventional group receiving daily interruptions in sedative infusions (4.9 versus 7.3 days and 6.4 versus 9.9 days, respectively).[32] In addition to trying to avoid or limit physical tolerance and withdrawal, successful tracheal extubation can be facilitated by avoiding residual effects of sedative agents which may impair upper airway control and respiratory function. To accomplish this, switching to agents which do not exhibit changes in their duration or effect following prolonged continuous infusions may also be beneficial. Therefore, it may be beneficial to switch to propofol or remifentanil for 8–12 hours prior to an anticipated attempt at tracheal extubation. As these agents do not exhibit a significant context sensitive half-life, especially remifentanil, their effects should dissipate rapidly upon discontinuation of the infusion. Given the potential for the development of the propofol infusion syndrome with the prolonged continuous infusion of propofol, this agent has been eliminated from the armamentarium of many PICUs.[33,34] However, the overwhelming majority of the severe cases of propofol infusion syndrome occurred with infusions of greater than 24–48 hours and, therefore, short-term infusions of 8–12 hours to allow for the clearance of the residual effects of other agents may be acceptable. However, it may be prudent to periodically monitor acid–base status and lactate levels even during the short-term administration of propofol and discontinue its administration immediately should a lactic acidosis develop as this may herald the onset of the other manifestations of the propofol infusion syndrome. Alternatively, the short acting synthetic opioid, remifentanil, may also be used to provide a deep level of sedation with a rapid offset once the infusion is discontinued. Remifentanil is metabolized by plasma esterases and demonstrates stable and similar pharmacodynamics across all age ranges.[18,35]

Its half-life of 8–10 minutes is constant even following prolonged administration and anecdotal experience has demonstrated that it may provide a deep level of sedation without residual effects when the infusion is discontinued.[35,36] The disadvantages of remifentanil which mandate its restriction to short-term (less than 24 hours) use are its cost and the rapid development of tolerance with the resultant need to rapidly increase the dose to maintain the same level of sedation.

Adjunctive agents for the perioperative care of these patients, such as dexmedetomidine, diphenhydramine, clonidine, phenothiazines, non-steroidal anti-inflammatory agents, acetaminophen, and chloral hydrate, have also been suggested by many authors to limit the need for opioids and benzodiazepines in an attempt to prevent or limit the development of tolerance, physical dependency, and withdrawal.[1,2,18] As these agents have limited effects on respiratory function, they may provide significant benefit particularly when used in rotation with opioids and benzodiazepines. Dexmedetomidine is an α_2_-adrenergic receptor agonist that possesses sedative, analgesic, and anxiolytic properties with no limited effects on respiratory function when administered within clinical dosing guidelines.[37,38] The short half-life of dexmedetomidine (~2 hours) allows easy titration by continuous infusion, quicker recovery, and fewer prolonged sedation related adverse effects. Given the concerns of respiratory depression, hemodynamic instability, and metabolic acidosis associated with the administration of propofol, dexmedetomidine may be a suitable alternative to allow for rapid tracheal extubation following prolonged sedation with opioids and benzodiazepines.

Although the majority of studies demonstrate a favorable pattern of hemodynamic stability of dexmedetomidine in pediatric patients, dexmedetomidine has the potential to produce dose-dependent decreases in blood pressure and heart rate.[39] Rarely, dexmedetomidine has also been reported to cause life-threatening complications including sinus arrhythmias, left ventricular dysfunction, refractory cardiogenic shock, and cardiac arrest.[37,40–42] Additionally, although approved for sedation during mechanical ventilation of adults, dexmedetomidine has not been approved by the Food and Drug Administration (FDA) for use in infants and children.[43]

Dexmedetomidine dosing regimens have been extrapolated from the adult literature, with modifications based on clinical experience in pediatric-aged patients. Current recommendations include a bolus dose of 0.5–1 µg/kg administered over 10 minutes, followed by a continuous infusion of 0.2–1.5 µg/kg/hour.[18] Dexmedetomidine has also been used during the extubation process to provide sedation and anxiolysis with limited effects on ventilatory function. It does not appear to significantly depress respiratory drive, thus interference with weaning from mechanical ventilation is less likely. In fact, it has been used both as a bridge to extubation and to expedite the process of weaning from mechanical ventilation.[44–46] In a study by Arpino *et al*., dexmedetomidine was initiated in a group of mechanically ventilated patients who failed previous attempts at weaning and tracheal extubation secondary to agitation.[44] With the administration of dexmedetomidine, 65% of the patients were able to undergo successful tracheal extubation. Dexmedetomidine was associated with a reduction in concomitant sedative and analgesic use with minimal adverse effects.

Non-steroidal anti-inflammatory agents and acetaminophen may be useful to provide adjunctive analgesia following these surgical procedures and thereby limit the use of opioid agents and their associated adverse effects. Although the non-steroidal anti-inflammatory agent, ketorolac, has been available for intravenous administration for years, recent additions to our practice include the availability of intravenous ibuprofen and the upcoming release of an intravenous acetaminophen preparation. Several studies in the pediatric population have demonstrated that these agents can effectively reduce opioid requirements by up to 20–30% following major surgical procedures. Additionally, the use of non-pharmacologic measures such as reduced stimulation, comforting, and regulation of day–night cycle are the other suggested methods to reduce the amount of sedative medications.[1]

## PHARMACOLOGICALLY INDUCED NEUROMUSCULAR BLOCKADE

Neuromuscular weakness due to the prolonged simultaneous use of corticosteroids and NMBAs can be another potential etiology for extubation failure in these patients. Although the theoretical benefit of corticosteroids is the reduction of post-surgical airway edema, our experience suggests that limiting the doses of corticosteroids to 24–48 hours in the peri-extubation period can be effective in reducing airway edema as well as preventing neuromuscular weakness and other adverse effects related to the more prolonged administration of corticosteroids. Although NMBAs may be needed in the immediate postoperative period to minimize movement of an indwelling ETT and disruption of suture lines, administration for more than 24 hours should be avoided if possible, given the risks of prolonged weakness and their negative impact on pulmonary toilet. Although seen most commonly in the adult population, there are anecdotal reports and an increasing awareness of such problems in the pediatric-aged patient. Potential techniques to avoid such complications include the use of intermittent boluses rather than a continuous infusion when NMBAs are administered,[2,3] monitoring with a nerve stimulator as is used in the operating room to guide the appropriate dosing of NMBAs, and discontinuation of the infusion on a daily basis to allow for the return of neuromuscular function and to evaluate whether ongoing neuromuscular blockade is still required. Even with appropriate monitoring, patients may have prolonged weakness after discontinuation of NMBAs. Prolonged recovery from NMBAs may be defined as a 50–100% increase in the time of recovery of muscle strength compared with that predicted by pharmacologic parameters after cessation of NMBA therapy, which is most often the result of accumulation of NMBAs or their metabolites.[18] We, therefore, recommend limiting the use of continuous infusion of NMBAs to the first 24 hours after the surgery with the transition to intermittent boluses after this period if ongoing neuromuscular blockade is deemed necessary.

### Tracheal secretions

Patients may also fail either a weaning attempt or an extubation attempt because of “excessive secretions”. ETT suctioning and chest physiotherapy (CPT) are traditional supportive elements in the care of children with “excessive secretions”. However, their routine use is not supported by evidence-based medicine, and in fact, some data suggest that these techniques may be detrimental. A set of papers describing the physiologic effects of CPT or ETT suctioning on paralyzed, sedated, mechanically ventilated children who had been deemed on assessment by the physical therapist to require CPT, demonstrated no benefit, and in one-third of the patients, the respiratory function deteriorated.[47,48] Some of the potential adverse effects of suctioning reported in the literature include hypoxemia, bacteremia, lobar atelectasis, mucosal trauma, hemorrhage, bronchoconstriction, lobar atelectasis, decreased cerebral oxygenation, pneumothoraces, cardiac arrhythmias, and cardiac arrest and even death.[47,48]

Commonly used suctioning systems are open-ET suctioning (OES) and closed system suctioning (CSS). CSS allows mechanical ventilation to continue during ET suctioning, and is most commonly used with an in-line multi-use suction catheter system encased in a plastic sleeve. Use of CSS may prevent ET suctioning induced hypoxia, decreases in lung volume, and spread of infection between patients and from patients to staff by limiting the spread of aerosolized infectious mucus particles.[49–52] It has also been suggested that CSS should reduce the risk of ventilator-associated pneumonia by eliminating environmental contamination of the catheter before introduction into the ETT.[52] However, there is paucity of evidence relating to the merits of CSS or OES in the pediatric critical care population. Some of the side effects of endotracheal suctioning may be minimized by reduction of suction pressure, limitation of the depth of insertion of the suction catheter, appropriate pre-oxygenation, adequate sedation and analgesia and muscle paralysis. We propose that suctioning should be performed only in cases of worsening pulmonary compliance (due to excessive secretions) and/or prior to extubation.

CPT has long been used with many other modalities as an adjunct to prevent and/or treat atelectasis and pneumonia in postoperative patients. However, CPT may have deleterious physiologic effects, especially in preterm and term neonates, as the literature has suggested its association with brain injury in very low birth weight infants[53] and severe hypoxemia in neonates.[54] In a prospective, randomized study that compared 19 patients who received CPT to 25 patients who did not receive CPT, CPT was associated with significantly more frequent and more severe atelectasis.[55] As such, CPT is not routinely recommended even during the use of NMBAs. Rather, our clinical experience suggests that the risks of respiratory issues can be lessened by adopting a ventilator strategy that provides for recruitment of atelectatic lung regions. The use of lower tidal volumes to prevent the theoretical risks of barotrauma in mechanically ventilated patients combined with the use of sedation and NMBAs prevents sighing, thereby leading to the development of progressive areas of atelectasis. To overcome such problems, we advocate the use of tidal volumes of 8–10 mL/kg, provided the mean airway and plateau pressures are kept within acceptable ranges, the application of 4–8 cm H_2_O of positive end expiratory pressure (PEEP), and the use of longer inspiratory times of 1–1.5 seconds. Additionally, intermittent sigh breaths can be delivered manually every 4–6 hours by disconnecting the patient from the ventilator and providing manual breaths with a resuscitation bag that is attached to a manometer so that a peak inflating pressure of 28–30 cm H_2_O is applied.

Although there is no evidence-based medicine to demonstrate the utility of prophylactic antibiotics in this select patient population, most centers advocate this practice until tracheal extubation is achieved. Additionally, given the risks of nosocomial infections in these patients, a high index of suspicion should be maintained with the use of frequent sampling of tracheal secretions for a gram stain and culture when infection is suspected due to temperature instability, worsening chest radiography, a deterioration in respiratory function, or an elevation of the white blood cell count.

## NUTRITION

Nutrition, either enteral or parenteral, should also be optimized to prevent neuromuscular weakness and facilitate the process of tracheal extubation. Enteral nutrition (EN) is the preferred route for critically ill children as it is more physiologic and is associated with decreased infectious complications and decreased length of hospital stay when compared with parenteral nutrition (PN).[56–59] Moreover, EN is also cost-effective and may have anti-inflammatory effects by lowering the expression of cytokines such as interleukin-6.[60] Furthermore, EN should be started immediately during the postoperative period as early feeding improves caloric intake and protein balance and in certain populations may even decrease mortality, without increased adverse events, when compared with EN delayed for 48 hours after admission.[61,62] Given the concerns of gastroesophageal reflux damaging the graft site, we would recommend, whenever feasible, to consider the use of postpyloric feedings with the use of promotility agents, proton pump inhibitors and/or H_2_-receptor antagonists. However, the available expertise and resources in individual PICUs may limit the placement of transpyloric tubes. The success of transpyloric tubes relies on the technique used, the experience and expertise of the operator, and the backup support from the radiologists in cases where bedside placement has not been successful. A variety of procedural techniques for transpyloric feeding tube placement have been described, but no single method has shown to be superior.[63–72] Despite the wealth of information regarding the techniques for placement of transpyloric feeding tubes and despite our current clinical practice of using such tubes, there are limited data from the literature to demonstrate any clinical advantages of such techniques when compared to gastric feeds through a nasogastric tube. In a multicenter, prospective, randomized single-blind study comparing the efficacy of gastrointestinal complications of early jejunal feeding with early gastric feeding in critically ill adults, it was found that gastrointestinal complications were less frequent in ICU patients fed into the jejunum.[71] However, EN using a nasojejunal route did not seem to be efficacious in decreasing nosocomial pneumonia in critically ill adults. Regardless of the site of enteral feedings, tube placement should be documented radiographically before initiating feeds. PN may be used to supplement or replace EN in those patients in whom EN alone is unable to meet the nutrition goals.

## NOSOCOMIAL INFECTIONS

Given their severity of illness and the need for prolonged endotracheal intubation and assisted ventilation, there may be an increased risk for nosocomial infections following LTR. Despite the lack of evidence regarding the use of prophylactic antibiotics, most centers advocate this practice until tracheal extubation is achieved. Additionally, given the risks of nosocomial infections in these patients, a high index of suspicion should be maintained with the use of frequent sampling of tracheal secretions and other instrumented sites (urine, central lines) for a gram stain and/or culture when infection is suspected due to temperature instability, worsening chest radiography, a deterioration in respiratory function, or an elevation of the white blood cell count. In an effort to decrease the risk of nosocomial infection, we would advocate the early removal or indwelling Foley catheters. Success of this practice can be facilitated by avoidance of excessive sedation and the administration of NMBAs which may increase the risk of such perioperative complications. Whenever feasible, avoidance of central venous cannulation is also suggested, given the potential risk of infectious complications. Avoidance of central venous cannulation can be facilitated by limiting perioperative laboratory evaluations and the use of EN.

## EXTUBATION READINESS

When a decision is made regarding an attempt at tracheal extubation, an evaluation of both the upper airway and respiratory function may be helpful in ensuring the correct timing of tracheal extubation. Air leak pressures around the ETT may provide an estimate of extubation success in these patients. Gustafson *et al*. demonstrated that patients without an air leak at 20 cm H_2_O or less were twice as likely to fail their initial attempts at tracheal extubation.[2] A pressure support trial or a T-piece trial prior to tracheal extubation may provide additional information regarding readiness for tracheal extubation, although conclusive data in children to support this approach are not available. It has been shown that successful extubation can be achieved equally effectively after a first breathing trial performed with pressure support of 10 cm H_2_O or breathing through a T-piece.[73] In their study of 257 subjects, Farias *et al*. found no differences in the rate of extubation failure within 48 hours (15.1% versus 12.8%) or failure of a spontaneous breathing trial (SBT) (20.8% versus 22.7%) in the two groups receiving either pressure support of 10 cm H_2_O or breathing through a T-piece for 2 hours. In another study by Chavez *et al*., an SBT was used for 15 minutes prior to extubation and consisted of providing a continuous flow rate (3 L/min for infants and 10 L/min for older children) via an anesthesia bag adjusted to provide a continuous positive airway pressure (CPAP) of 5 cm H_2_O.[74] Of the 70 patients, 64 passed (91%) and, of these, 5 subsequently failed extubation (7.8%) (one reintubation, four required noninvasive ventilation). The failed extubation rate was no better than historical rates where extubation was based on clinical decision alone. Although the SBT had high sensitivity (95%) and positive predictive value (92%), the high success rate could have been simply because all the patients enrolled in the study were deemed ready for extubation by the clinicians. In essence, the SBT did not contribute to predicting a successful extubation compared to clinical decision alone. However, in a recent systematic review on weaning and extubation readiness for pediatric patients, Newth *et al*. proposed that a CPAP or T-piece trial is better than the use of pressure support with PEEP, as adding pressure support is likely to mask respiratory insufficiency and contribute to a higher failure rate following tracheal extubation.[75] They argue that if an infant or young child cannot sustain an SBT on CPAP or a T-piece for several hours, they are likely to fail extubation.

Measurement of the negative inspiratory force (NIF), also referred to as maximum inspiratory pressure, has also been found to be a predictor of tracheal extubation success in adults.[76,77] However, Venkataraman *et al*. did not find this test useful as a single test predictor for extubation success.[78] Newth *et al*. concluded that NIF of –30 cm H_2_O is probably reassuring in a spontaneously breathing patient for extubation readiness, but is unreliable and not validated in children and should be applied with caution.[75]

## CONCLUSIONS

Single-stage or two-stage LTR is a complicated airway reconstructive surgery that requires meticulous postoperative care in an experienced PICU to ensure success. Timely tracheal extubation is the key to success in these surgeries and extubation failure in these patients can lead to reintubation, surgical failure, prolonged PICU care, and in some cases, the need for a tracheostomy to maintain an adequate airway. Younger patients can be extremely challenging in the postoperative period as the literature has demonstrated a higher failure rate in many aspects of their postoperative care. We propose the following measures to improve the success and limit the morbidity following LTR:

- nasotracheal intubation whenever feasible,
- earlier weaning of opioids and/or benzodiazepines with consideration of transition to short-acting agents (propofol or remifentanil) or dexmedetomidine for 8–12 hours prior to tracheal extubation,
- avoidance of excessive dosing of sedative and analgesic agents by following pediatric pain scores,
- use of non-pharmacologic measures such as reduced stimulation, comforting, and regulation of day–night cycle to minimize sedation,
- use of adjunctive medications as clonidine, diphenhydramine, phenothiazines, non-steroidal antiinflammatory agents, and acetaminophen for sedation and analgesia,
- use of dexmedetomidine during the tracheal extubation process,
- avoidance of simultaneous prolonged administration of neuromuscular blockade and corticosteroids,
- use of intermittent rather than continuous neuromuscular blockade, if necessary, with monitoring by use of a train-of-four device,
- optimizing caloric intake from the first postoperative day with an emphasis on aggressive use of the enteral route,
- limitation of the risk of nosocomial infection by avoidance of central venous cannulation and early removal of urinary catheters,
- maintenance of a high index of suspicion for the presence of nosocomial infection with sampling of blood, urine and tracheal secretions for signs suggestive of infection, including temperature instability, elevated white blood cell count, worsening respiratory function or changes on a chest radiograph,
- testing air leak pressures around the ETT prior to tracheal extubation,
- a CPAP or T-piece trial prior to extubation or any other accepted method of testing readiness for extubation and
- effective communication among team members and the families.
